# A Survey of the Transcriptomic Resources in Durum Wheat: Stress Responses, Data Integration and Exploitation

**DOI:** 10.3390/plants12061267

**Published:** 2023-03-10

**Authors:** Diana Lucia Zuluaga, Emanuela Blanco, Giacomo Mangini, Gabriella Sonnante, Pasquale Luca Curci

**Affiliations:** Institute of Biosciences and Bioresources, National Research Council (CNR), Via Amendola 165/A, 70126 Bari, Italy

**Keywords:** RNA-seq, gene expression, environmental stresses, stress resilience, climate change

## Abstract

Durum wheat (*Triticum turgidum* subsp. *durum* (Desf.) Husn.) is an allotetraploid cereal crop of worldwide importance, given its use for making pasta, couscous, and bulgur. Under climate change scenarios, abiotic (e.g., high and low temperatures, salinity, drought) and biotic (mainly exemplified by fungal pathogens) stresses represent a significant limit for durum cultivation because they can severely affect yield and grain quality. The advent of next-generation sequencing technologies has brought a huge development in transcriptomic resources with many relevant datasets now available for durum wheat, at various anatomical levels, also focusing on phenological phases and environmental conditions. In this review, we cover all the transcriptomic resources generated on durum wheat to date and focus on the corresponding scientific insights gained into abiotic and biotic stress responses. We describe relevant databases, tools and approaches, including connections with other “omics” that could assist data integration for candidate gene discovery for bio-agronomical traits. The biological knowledge summarized here will ultimately help in accelerating durum wheat breeding.

## 1. Introduction

In the natural environment, plants often face unfavorable factors affecting their growth and production. Especially in recent years, the effects of climate change on the future of agriculture are particularly crucial, since crops are experiencing unprecedented climate conditions. Plant stress deriving from adverse environmental events includes biotic and abiotic stress. Biotic stress is due to pathogens such as viruses, bacteria, fungi, nematodes, and parasitic plants. Abiotic stress can be caused by one or more physical or chemical factors, including drought, high salt concentration, high or low temperature, nutrient limitations, heavy metals, etc. Plants cope with hostile environmental stimuli by activating a cascade of responses at various (cellular, organ, physiological, biochemical, molecular) levels, ultimately leading to phenotypic changes. These reactions have not been fully understood yet, and a myriad of studies have been published in the last decades to increase knowledge in this field, including investigations carried out at the transcriptomic level. The transcriptome represents the complete set of all RNAs (mRNA and non-coding RNA) transcribed by a particular cell (or tissue, organ) in a precise developmental stage or physiological condition. Transcriptomics is the study of the transcriptome, and includes RNA transcription and expression levels, functions, locations, trafficking, and degradation. It also comprises the structure of transcripts and their related genes, in terms of their start sites, 5′ and 3′ ends, and post-transcriptional modifications [1]. Currently, microarrays and RNA-seq, developed in the mid-1990s and 2000s, are the two main techniques that allow measurement of expression profiles of thousands of transcripts at the same time. The former quantifies the abundances of a preselected set of transcripts by their hybridization to an array of complementary probes, while the latter, developed more recently, leverages high-throughput sequencing technologies to measure all transcript cDNAs [2]. 

Durum wheat (*Triticum turgidum* subsp. *durum* (Desf.) Husn.) is a tetraploid (genome BBAA) cereal crop originating in the Fertile Crescent from the domesticated emmer wheat (*T. turgidum* subsp. *dicoccum* (Schrank ex Schübl.) Thell.), which is in turn derived from the wild emmer wheat (*T. turgidum* subsp. *dicoccoides* (Körn. ex Asch. & Graebn.) Thell.) [3]. Durum wheat, mostly used for pasta production, is mainly cultivated in the Mediterranean countries, North America, and Australia. The European Union is the major producer in the world and Italy is the principal European country, followed by France and Turkey (International Grains Council, https://www.igc.int/en/default.aspx, accessed on 10 May 2022). Some regions devoted to durum wheat cultivation, e.g., southern Europe, northern Africa and the Horn of Africa, are characterized by low-rainfall and high temperatures, with critical drawbacks such as drought, salinity and low inorganic matter, often resulting in growth limitations and low yields. It is then important to investigate and elucidate the molecular basis involved in traits responsible for the adaptation and resilience of this crop to climate changes, along with other challenges of modern agriculture. 

Transcriptomic analysis for durum wheat historically relied on bread wheat genome data leveraging the Affymetrix GeneChip^®^ Wheat Genome Array for microarray technology applications, followed by a massive transition to RNA-seq technology after the publication of the genome reference for the hexaploid wheat variety “Chinese Spring” [4]. Subsequently, the durum wheat genome has been sequenced [5] and could finally be used for reference-based RNA-seq analysis. Despite the large and increasing number of transcriptomic datasets available and annotated on public repositories, metadata (e.g., species names, study types, project identifiers, etc.) are still often missing or inconsistent in and across repositories and publications. This irregularity can represent a limit to the generation of novel scientific conclusions through large scale biological studies, which require appropriate data and metadata for collection and classification of transcriptomic datasets. In this review, we summarize the public transcriptomic resources available for durum wheat, with a special focus on those addressing plant stresses. We highlight the main results, describe where these datasets can be found, and ultimately delineate comparative and integrative approaches to the exploitation of public transcriptomic resources in order to drive novel scientific knowledge.

## 2. Bibliometric Insights on Tetraploid Wheats and Focus on Publicly Available Transcriptomic Resources for Durum Wheat

**Figure 1:**
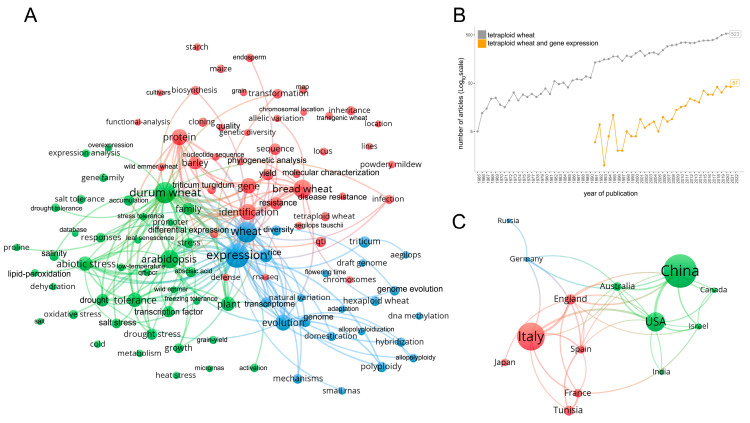
Research topics and their evolution over the years of publications on tetraploid wheat with a focus on gene expression. (**A**) Term map for the 532 retrieved publications focused on gene expression where different colors (red, green, blue) represent the terms (keywords) belonging to different clusters. The size of the nodes (circles) is based on the number of occurrences. Links between nodes indicate the co-occurrence of terms. The connecting lines indicate the 200 strongest occurrence links between terms (minimum strength = 7). (**B**) Number of publications over time on tetraploid wheat subspecies (grey color) and the subset of 532 publications focused on gene expression (orange color). (**C**) Term country map representing country collaborations for the 532 retrieved publications (minimum number of documents per country = 15). The size of the connecting lines indicates the strength of the co-occurrence (minimum strength = 2).

A bibliographic analysis was carried out with the idea of surveying the research trends on gene expression studies on durum wheat and the other tetraploid wheats, gathering information on the development of various research areas over time and identifying the most recurrent keywords. The Web of Science (WoS) database was used on 15 January 2023 to retrieve bibliographic records related to gene expression studies in tetraploid wheat [6] by using the string “(*Triticum* OR wheat) AND (*durum* OR *turgidum* OR *carthlicum* OR *dicoccum* OR *paleocolchicum* OR *polonicum* OR *turanicum* OR *dicoccoides* OR *timopheevii* OR *armeniacum* OR tetraploid*) AND (gene expression)” in the combined fields of title and abstract, to include information on all tetraploid wheat subspecies. No starting date was given, thus allowing the search database to find the earliest articles in the literature. This search resulted in 532 publication records, of which ~60% were focusing on durum wheat, as resulted from a similar search performed with the substring “(*Triticum* OR wheat) AND (durum)”. The tetraploid set of retrieved publications was used as input in the VOSviewer software (version 1.6.18.0; [7]) to create bibliometric maps. Term maps are two-dimensional representations of a research field produced using VOSviewer. The cluster analysis of terms related to the field present in the title and abstract of the retrieved 532 publications published in the period 1991–2022 is illustrated in Figure 1A. A total of 105 keywords, with a minimum number of occurrences of eight, are grouped in three main clusters (each with a minimum of 20 keywords), which provide an overview of the structure of the research themes (Figure 1A). The red cluster mainly includes terms related to wheat genetics, addressing research on variety and species diversity. The terms “QTL (quantitative trait loci)”, “yield”, “resistance” and “quality” are mainly recurrent in recent publications (2016–2022), highlighting the modern research trend for discerning traits for wheat productivity. “Inheritance”, “phylogenetic analysis”, “identification” and “expression” of genes and proteins are topics in common across both early and late publications, underlining the common thread of identifying and mapping molecular variants. “Biosynthesis”, “endosperm” and “grain” are also recurrent terms, as genetic and biochemical studies are included in this cluster. “Disease resistance” and “infection” are also reported research terms. The blue cluster contains keywords inherent to gene expression, genome evolution studies, adaptation and domestication. The green cluster comprises the more recent transcriptomic studies, exploiting gene expression patterns with a specific view on abiotic stresses, such as salt, drought and heat. However, the three clusters are tightly interconnected because some aspects of wheat research, such as “expression”, “wheat”, “evolution”, and “stress”, can be included in more than one cluster (Figure 1A). Overall the scientific interest and production on tetraploid wheat expression data is growing and this is testified by the increasing number of publications over time on gene expression studies in tetraploid wheat species, showing an average yearly growth rate more than three times greater compared to all published papers on tetraploid wheat (obtained using the same previously mentioned search string, but excluding “gene expression”, to capture broad-spectrum research on tetraploid wheat) in the period 2000–2022 (17.8% versus 5.3%) (Figure 1B). The clustering of country collaborations, represented by the term country map in Figure 1C, shows that the main countries involved in durum wheat expression studies form three groups, where China, Italy and the USA publish, in this order, the majority of studies. China leads the green cluster, with the USA, Canada and Australia as the main collaborators. Italy is the major country in the red cluster, working in collaboration with other European countries such as England, Spain, France and with Tunisia (Figure 1C). This map highlights the importance, in terms of scientific contribution, of the Mediterranean countries, reflecting the importance of wheat for their agriculture and food sectors. 

As one of the main goals of this review was to summarize the publicly available transcriptomic resources available on durum wheat, we screened the Sequence Read Archive (SRA) database with the following string “(“*Triticum turgidum*”[Organism]) AND (“mirna seq”[Strategy] OR “ncrna seq”[Strategy] OR “other”[Strategy] OR “rna seq”[Strategy]) AND (“other”[Source] OR “transcriptomic”[Source]) AND (“study type other”[Properties] OR “study type transcriptome analysis”[Properties] OR “study type transcriptome sequencing”[Properties])”. The broad search, including as organism the species “*Triticum turgidum*” and, as strategy, source, and properties, the term “other”, was also motivated by the idea to retrieve database records with potentially too general, inconsistent or missing metadata. The search output included, as expected, the majority of the records (>70%) on *Triticum turgidum* ssp. *durum*, and was filtered by manual inspection to confirm that durum wheat was included as an organism under investigation in the study, and to be sure to link each record to a paper published by the end of 2022 (full list available in Table S1). This search was integrated with other records from a parallel search on the ArrayExpress database and with a manual inspection of the collected 532 papers published by the end of 2022 filtered for those generating transcriptomic resources on durum wheat. This screening resulted in 62 publication records with available transcriptomic data, classified on the basis of several features (e.g. genotype, tissue, treatment, etc.). This information is available in Table S1, where we connected each reference ID mentioned in original publications to an SRA-BioProject. A sub-table focusing on plant stresses was extracted and is summarized in Table 1.

## 3. Biotic Stresses

Crop production losses due to pests increase globally with rising temperatures in all climate models and across all biological parameters. When average global surface temperatures increase by 2 °C, the median increase in yield losses owing to pest pressure is 46% for wheat [42]. Most plant-breeding programs target developing genotypes, which are resistant to plant pathogens so that crop loss due to biotic stress could be mitigated [43]. A prerequisite to achieve resistant cultivars (cvs) to biotic stress is to identify resistance genes and to investigate molecular changes in response to pest attack. These goals can be obtained using transcriptomic approaches although, in durum wheat, they have been applied limitedly to fungal diseases and insect attacks.

### 3.1. Fungal Diseases

In the last 20 years, *Fusarium* head blight (FHB) caused by *Fusarium graminearum* has induced significant yield loss and the production of mycotoxins such as deoxynivalenol (DON) that can accumulate in the kernels. The comparisons of transcriptomic profiles between FHB resistance and susceptible genotypes are used to evaluate the gene expression variation in response to *F. graminearum* attack. Five expression networks related to the response to FHB were identified using a resistant Persian wheat line (REB6842), a susceptible durum cultivar (Strongfield) and two transgressive double haploid (DH) lines [8]. Two networks were different among these genotypes but similar between inoculated and mock-inoculated. This suggested that genes in these networks may be involved in constitutive defense mechanisms. The remaining three networks showed differences between treatments, indicating that the FHB resistance involved inducible defense genes. Functional annotation clustering of expression networks identified different gene clusters in multiple networks having nucleotide binding (NB-ARC), leucine-rich repeat (LRR), F-Box, FAR1 and Zn finger, and protein kinase domains. Among the hub genes identified, NBS-LRR genes such as the orthologues of *RPP8*, *RGA2*, *RGA4* and *At3g14460,* were more expressed in the susceptible compared to the resistant genotypes. ABA signaling pathway hub genes including *HAB1*, *UBA2a*, and *SRK2E* and transcription factors such as *MADS* members were also identified as candidate genes [8]. 

Genes encoding NB-ARC domain-containing disease resistance proteins and LRR receptor kinases were also found to be differentially expressed in a resistant line obtained with a DNA methylation inhibitor and the susceptible parental line by Kumar et al. [9]. In this experiment, genes differentially expressed in response to *Fusarium* infections were related to signaling pathways, involved in the biosynthesis of secondary metabolites related to oxidative activity, hormone signaling, while several genes were transcription factors (*bZIP*, *AP2/EREBP*, and *WRKY*), or cell wall and pathogenesis related (PR) genes. A panel of differentially expressed genes (DEGs), including DNA binding proteins, FAR1, protein kinases, and bZIP transcription factor, were differentially expressed in a RNA-seq experiment performed on a susceptible durum cultivar (Langdon) and a resistant disomic substitution line (Langdon-*T*. *turgidum* subsp. *dicoccoides*) [10]. These results confirmed that the modulation of these genes might contrast with pathogen progression and therefore reduce the extent of disease spreading.

Another important fungal disease for foliar damage and losses in yield in several durum cvs growing areas is the powdery mildew, caused by the pathogen *Blumeria graminis* (DC) Speer f. sp. *tritici* Em. Marchal (*Bgt*). In order to characterize the powdery mildew resistance gene *Pm68*, Bulk Segregant RNA-seq (BSR-seq) analysis was performed [11]. The experiment was performed on a F_2_ population derived by crossing two durum accessions (TRI1796 and PI584832) polymorphic for response to virulent isolate BgtYZ01. Over forty thousand SNPs and InDels were identified and, among them, 42 SNPs were enriched in an approximately 8.6-Mb region (16.2–24.8 Mb) on the short arm of chromosome 2B, where *Pm68* was mapped.

A transcriptomic approach was used to identify DEGs related to ergot resistance in DH lines contrasting in response to *Claviceps purpurea* (Fr.) Tul. obtained by a strong ergot resistance recombinant inbred line (RIL) and a susceptible durum cultivar (Avonlea). A total of 351 DEGs were found that were mapped on chromosomes 1B (35%), 2A (21.4%), 5A (7.1%), and 5B (18.8%). Interestingly, in these chromosomes, 114 out of 351 DEGs were co-localized within QTLs for ergot resistance. MYB transcription factors (TFs), F-box and ankyrin repeat-containing protein, BTB/POZ-containing proteins and protein kinase were the DEGs of major interest. In particular, the detection of MYB TFs suggested that the *C. purpurea* infection can induce a significant change in the expression of genes involved in hormonal pathways, such as gibberellic acid (GA) [12].

Integration of transcriptomic data on biotic stress in durum wheat allowed understanding of the essential role of epigenetic processes in the regulation of the transcriptomic response to fungal infection. The multidisciplinary approach also unveiled the mechanism involved in pathogen resistance conferred by specific QTL.

### 3.2. Insect Attacks

The major wheat insects can be considered as the aphids, which can cause direct damage to plant development and are responsible for the transmission of plant viruses. In response to aphid attacks, plants can perceive damage-associated molecular patterns (DAMPs) or aphid-associated molecular patterns (AAMPs) and subsequently induce a plant defense response. This defense consists in the production of specialized metabolites often acting as feeding deterrents, reducing damage from aphids. Among these, the benzoxazinoids (BXDs) are abundant in cereal species such as wheat, where their levels are tissue and genotype dependent [44]. The total transcriptome and metabolome of three wheat genotypes (wild emmer, durum wheat, and bread wheat) were used to study defense against aphids [13]. The insect attack was performed, confining ten adult *Rhopalosiphum padi* aphids on the second leaves of 11 day-old plants for 6 h. The analysis showed a unique transcriptome pattern for each genotype. The DEG analysis identified almost 9000 unique transcripts devised in eight clusters split into five expression groups. In particular, the gene expression of specific biosynthetic genes of the benzoxazinoid pathway resulted in differences between the domesticated genotypes (durum and bread wheat) and wild emmer. High expression levels were found in wild emmer for genes such as *P450*, *Bx3*, *Bx5*, and in durum and bread wheat for downstream glucosidases and O-methyl-transferases.

Lepidopteran attacks such as *Spodoptera littoralis* produced a strong variation of transcriptome and BXDs metabolites. Indeed, the transcriptomic analysis revealed that several *Bx* genes were significantly up-regulated in response to caterpillar feeding, including indole-3-glycerol synthase (*IGPS1* and *IGPS3*), *Bx2*, *Bx3*, *Bx4*, *Bx8/9*, and *Bx10*. In addition, both for aphids and caterpillars, the expression level of the dioxygenase *Bx6* significantly increased and was higher upon caterpillar feeding in comparison to the untreated controls [14]. Finally, by exploiting the transcriptomic dataset, the up-regulation of the two transcription factors *MYB31* homoeologous genes was observed upon aphid and caterpillar feeding [14]. In another study, a large panel of DEGs including genes such as *PAL*, *PIs*, *PR1*, and *LOX* and gene families related to ethylene (ET) signaling pathways were up-regulated in a resistant wheat line after corn leaf aphid (*Rhopalosiphum maidis*) attack when compared with the susceptible durum line [15]. The multi-omics approach provided insights into the important role of chemical mechanisms as mainly responsible for an efficient defense of durum wheat against aphids. The studies on biotic stresses demonstrate that the activation of defense systems, through the modulation of the transcription of specific durum wheat genes, generates resistance to pathogens. This knowledge can help the development of new durum wheat varieties with improved resistance to fungal diseases and insects.

## 4. Abiotic Stresses 

### 4.1. Heat and Water-Deficit Stress

Global warming is becoming a serious problem for crop growth and yield. Worldwide agricultural production is highly affected by heat and drought stresses, particularly in the Mediterranean Basin. An increase in ambient temperature negatively affects wheat grain production by increasing photorespiration and inhibiting photosynthesis, promoting early senescence, and stunting plants [45,46]. Water-deficit stress frequently occurs in durum wheat during the reproductive stage, resulting in a negative impact on grain-setting processes, such as floral initiation, fertilization and spikelet development, but it can also continue during the grain filling stage, thus affecting biological processes related to dry matter accumulation, such as nutrient transport and photosynthesis [47]. As a consequence, there is a decrease in grain yield and quality, especially protein content and carbohydrate composition, which are directly associated with pasta-making and other uses [47]. 

Heat and water-deficit stress notably modulate the photosynthetic activity in durum wheat. A lower expression of transcripts involved in photosynthesis and reconfiguration of primary and secondary metabolism after water-stress was observed in three breeding lines with different yield stability after water stress [16]. On the opposite side, under heat stress, the transcriptional response of durum wheat seedlings showed an over-representation of genes related to photosynthetic acclimation, respiration, lipid biosynthesis, carbon balance, ROS regulation, and physiological progression acceleration [24]. In addition, an analysis of flag leaves of plants exposed to high temperature displayed an up-regulation of the expression for the ribulose-1,5-bisphosphate carboxylase oxygenase (Rubisco) large subunit and for Rubisco activase genes, while a down-regulation of genes involved in light reactions, carbohydrate metabolism, glycolysis, mitochondrial electron transport, nitrogen (N) metabolism, amino acid metabolism, cell modification and secondary metabolism was observed [32]. In grains at the early filling stage, short-term heat stress produced an initial transcriptomic response characterized by the induction of chaperones and genes associated with photosynthesis and the inhibition of genes involved in carbohydrate metabolism [25]. Moreover, the molecular response to drought can be influenced in durum wheat plants with alteration in pigment content by genes encoding antioxidant enzymes, photosystem components, and enzymes representing carbohydrate metabolism and the tricarboxylic acid cycle [17].

Multi-omics analysis using next-generation sequencing has been carried out to study the water-deficit and heat stress response in the stress-tolerant DBA Aurora, a leading Australian commercial variety, and the stress-sensitive L6 breeding line at the whole genome level [27,28,29]. Integrated analysis of small RNA, transcriptome, and degradome sequencing was applied during reproduction in flag leaves [29], identifying DEGs linked to processes associated with hormone homeostasis, photosynthesis and signaling, and in developing grains [28], where observed DEGs were enriched in nutrient metabolism, cellular differentiation, transport, reproductive development, and hormone transduction pathways. In addition, the study of the microRNAome at the young seedling stage revealed the transgenerational effects of water-deficit and heat stress in the same genotypes [27]. In particular, the study revealed significant differences in miRNA expression on the seeds of DBA Aurora and L6 plants previously treated with both stresses at the booting stage. Pathway enrichment analysis showed miRNA target genes to be associated mainly with starch and sucrose metabolism and MAPK signaling pathways. The stress-tolerant DBA Aurora genotype and DBA Artemis, another high-yielding Australian durum wheat variety, were also compared. Small RNA, transcriptome and degradome sequencing data integration, showed transgenerational effects of water-deficit and heat stress in these durum wheat genotypes [18,26]. Effects of water-stress in parental and progeny plants of the Australian durum genotypes showed significant biological interactions between water stress-responsive miRNAs and targets, with transgenerational stress tolerance potentially generated through hormone signaling and nutrient metabolism pathways [18]. On the other hand, heat stress treatment applied to the parents produces a positive effect on the progeny plants by increasing chlorophyll content, grain weight, grain number and grain total starch content [18]. Integrated sequencing analysis, GO and pathway analysis suggested that stress responsive miRNAs and their target genes have functional roles in hormone homeostasis, signal transduction and protein stabilization [26]. In a different study, the characterization of the miRNAome of durum cv. Simeto highlighted microRNAs differentially modulated by early water stress conditions, with target genes annotated as transcription factors including NAC, genes involved in the synthesis of phenylpropanoids, oxidases, and lipid binding-proteins [19].

Transcriptomic analysis of drought stress response in roots of the modern durum cultivar Kiziltan supported the existence of lncRNA–miRNA–mRNA networks and drought-responsive transcripts modulating biological processes such as oxidation-reduction and protein phosphorylation [20]. Moreover, the miRNA target genes identified were involved in metabolic processes with functions ranging from tRNA processing and branched-chain amino acid biosynthesis to proteasomal protein and toxin catabolic processes [21]. One of the ESTs identified, a target of miR1137, was annotated as a glutathione S-transferase, suggesting that miR1137 is a key player under drought stress conditions in modern wheat [21]. Root transcriptomic profiling was obtained from a introgression tetraploid line from the wild emmer (Zavitan), which exhibited a significant differential root growth and shift in root-to-shoot ratio in response to drought stress, compared to durum cultivar Svevo [22]. Combining transcriptomic and genotypic data, DEGs were mapped to specific introgressions identifying a candidate gene encoding for a root-specific kinase, which may be linked to the root-to-shoot carbon reallocation under drought stress conditions.

Differences in the drought stress response of durum and bread wheat at the grain filling stage have been observed, highlighting a substantially different molecular response between both species, with genes involved in ABA, proline, glycine-betaine and sorbitol pathways being up-regulated by drought stress [23]. In a later study, two opposite drought and heat stress-responsive strategies were observed in the durum wheat cultivars Ofanto and Cappelli [30], in agreement with the contrasting differences in water use efficiency (WUE) and stomatal conductance (SC) that these genotypes displayed [48]. 

The studies mentioned in the present section analyze the transcriptome of durum wheat plants after heat and/or drought stresses to decipher the molecular mechanisms involved in the thermal and drought adaptation in durum wheat. In many cases, comparative transcriptomic studies were carried out, including durum wheat genotypes with varying stress tolerance profiles, which represents a powerful tool for discovering genes involved in drought and heat tolerance. DEGs found between tolerant and sensitive genotypes represent candidate genes to be characterized and allele mined with the aim of identifying useful alleles to accelerate breeding programs, with the purpose of improving drought tolerance and crop yield in a future high temperature and drought scenario. Some studies showed a complete transcriptomic profile that includes mRNA transcriptome, small RNA-ome, and mRNA degradome, while other works integrated transcriptomic data with genomics, proteomics, phenomics or e-QTL analysis. However, more multidisciplinary studies, including metabolomics, will be necessary to obtain an effective strategy to understand gene functions, genome structures, biological pathways, metabolic and regulatory networks underlying heat and drought stress adaptation in durum wheat.

### 4.2. Nitrogen Stress

Crop productivity depends on the availability of N in the soil, since this macronutrient affects fundamental plant processes, such as growth, architecture, flowering, senescence, photosynthesis and grain yield [49]. In fact, N is essential for the plant cell machinery, being used for the synthesis of nucleic acids, amino acids, proteins and several hormones [50]. Cereals, and wheat in particular, are highly demanding crops in terms of N, and high quantities of N fertilizers are used worldwide each year. However, the production and use of chemical fertilizers poses serious environmental concerns, and for a more sustainable agriculture, the development of plants with a higher N use efficiency is desirable [51]. Therefore, it is necessary to study plant adaptation to low N environments and understand the molecular mechanisms underlying plant responses to low N.

Nitrogen metabolism in plants involves several phases, including N uptake and regulation, N reduction and signaling, amino acid metabolism and transport, interactions between N and carbon metabolism, N translocation and remobilization [52,53]. Low and high affinity nitrate transporters (NRT1 and NRT2) are used by the plants to uptake nitrate from the soil. Nitrate is subsequently reduced to nitrite and to ammonium. The ammonium resulting from nitrate or directly taken up by ammonium transporters is then integrated into amino acids, mostly through the enzymes glutamine synthetase and glutamate synthase in plastids [54].

In durum wheat, plants grown under chronic N starvation conditions have shown several phenotypic/physiological changes, such as chlorotic leaves, shortening of the vegetative phase, with acceleration of plant flowering time and senescence, as well as reduced tiller number, flag leaf area, number of spikelets per spike, and kernel weight per spike [33,55]. A transcriptomic analysis of different organs from these stressed plants (cv. Svevo) resulted in over 4600 DEGs, especially in roots, followed by stem/leaf, flag leaf, and spike tissues. The most commonly represented biological processes involved were N compound metabolism, carbon metabolism, and photosynthesis [33]. Among the DEGs identified, many genes were involved in N absorption and assimilation, including nitrate or ammonium transporters, Nitrate reductase [NADH] 1, Asparagine synthetase, Glutamine Synthetase, Glutamate synthase 1 [NADH-GOGAT 1], and Glutamate dehydrogenase. Genes deputed to the transport of other nutrients were also found differentially expressed, such as potassium, phosphate, iron, sugar, ABC transporters, etc. [33]. Due to the strong relationship between N and carbon (C) metabolisms, N stress led to a general modification in the expression of genes participating in glycolysis, photosynthesis, photorespiration, etc., which are involved in carbon metabolism. Moreover, the comparison between transcriptomic and metabolomic response of durum and emmer wheat to N starvation highlighted a broader and stronger response in durum wheat [34].

Several conserved, as well as novel, miRNAs have been found to be differentially expressed in durum wheat plants following N stress, often differing in time, space, and genotype [35,36,56]. For instance, the complex spatial regulation of ttu-miR164 and ttu-miR167 in the roots of cv. Svevo, divergent from cv. Ciccio, might be related to a specific root architectural adaptation to N stress [35]. Another miRNA with a different expression in the two cultivars is ttu-miR827, possibly involved in phosphate absorption by targeting nitrogen limitation adaptation (NLA), as observed in other species [57,58]. A strong down-regulation of the ttu-miR169 family members (ttu-miR169c and the ttu-novel-61) was observed in Svevo and Ciccio plants during N-stress at the grain filling stage, and experimental data indicate that the target of ttu-novel-61 is *CCAAT-TF WHAP6*, a gene possibly playing a role in the N response adaptation in long-term stress [35,36]. Another study [37] provided a description of the durum wheat miRNAome with particular expression patterns subject to the progeny N stress, parental treatment, and genotype factors, together with the annotation of their functional target genes. The analysis of physiological, morphological, and molecular responses of Australian durum wheat to N starvation under the influence of parental water-deficit and heat stress suggested that the cross-stress impacts of transgenerational stress are genotype-dependent [37].

Scientific works studying the transcriptomic response to nitrogen starvation in durum wheat showed DEGs and microRNAs in specific genotypes using different tissues and developmental stages. Integration of these data in a miRNA-mRNA combined analysis would be useful to clarify the targeted regulatory relationship between the single miRNAs and mRNAs, giving deeper insights into durum wheat responses to this nutritional stress. Considering the importance of root tissue to uptake water and nutrients from the substrate, root system should be essential in the adaptation of durum wheat to nitrogen stress as was suggested by the high number of genes and miRNAs differentially expressed under N stress in this tissue [33,35,36]. Data integration with high-throughput phenotyping of roots hidden underground would provide an in-depth characterization of the N stress response including root system architecture data from a large number of durum plants without sampling and washing roots, obtaining more information for breeding programs aiming at developing climate-resilient durum wheat plants.

### 4.3. Other Abiotic Stresses

Transcriptome analyses help in unraveling molecular dynamics and players of the complex plant responses to many other abiotic stresses such as salinity, metal ion toxicity, extreme temperatures, oxidative and hyperosmotic stresses, which influence plant development and important traits such as yield, quality, and security of wheat-based products.

Crop productivity is critically reduced by soil salinity, causing plant osmotic stress and ion toxicity [59]. The challenge is to gain quantitative data for the role of specific salt tolerance mechanisms at the genetic level, as molecular markers for salinity tolerance. RNA-seq could serve as an efficient strategy for developing SNP markers for durum wheat cultivars, as recently demonstrated by a study addressing the transcriptomic responses to salinity treatment of two Tunisian cultivars showing different degrees of salinity tolerance [38]. Gene expression in leaf blades was analyzed using RNA-seq and 17 SNP markers were developed, based on the RNA-seq data of commonly up-regulated genes. Twenty-six accessions of Tunisian durum wheat were subjected to salinity stress and three SNPs were present only in salinity-tolerant lines but not in moderately tolerant or susceptible lines [38], thus demonstrating that RNA-seq is an efficient tool for identifying SNPs in the complex genome of durum wheat. The transcripts harboring these three SNP markers encode the protein FAR1-RELATED SEQUENCE 12 and the mitogen-activated kinase YODA, both associated with adaptations to environmental and salinity stresses [38].

Another relevant stress is represented by Cadmium (Cd), one of the most toxic heavy metals, both for human health and for plants [60]. Wheat, in particular, is highly vulnerable to Cd toxicity as it tends to accumulate Cd in the grain [61], thus affecting human health. As in other plant species, comparative transcriptomic studies on wheat roots revealed a multilevel coordination of Cd uptake, transport, sequestration and detoxification, both in durum [39,40] and bread wheat [62]. Recently, the transcriptomes of roots from two commercial durum cultivars and from two near isogenic lines with contrasting Cd accumulation in grains were analyzed [39,40], identifying the key genes and mechanisms involved. The mRNA sequencing of Creso and Svevo durum cultivars in response to Cd treatment identified about 7100 DEGs in root and shoot tissues, revealing a conserved strategy to detoxify cells from Cd [39]. Four main interconnected responses were depicted, in the light of the regulated contigs, with high similarity to the sequences of Arabidopsis genes: the activation of transcription factors, principally *WRKY* and *bHLH* families, the latter probably responsible for the strong up-regulation of three nicotianamine synthase genes (*NAS2*, *NAS3*, and *NAS4*), which sustain the production of the phytosiderophore nicotianamine (NA) able to chelate heavy metals. An up-regulation of the methionine salvage pathway was also found, tightly connected with NA biosynthesis. Moreover, the vacuolar *ZIF1* and *ZIF-*like genes, and the *YLS1* and *YSL2* genes, which are all NA chelating heavy metal transporters, were also strongly activated [39].

The elucidation of stress response mechanisms guide the development of breeding strategies or genetic modifications to obtain cultivars that do not translocate Cd up to the grains [63]. The transcriptome of roots of two Cd-treated or untreated low-Cd and high-Cd near-isogenic lines of durum wheat, accumulating Cd in grains, was carried out to reveal elements differentiating the low-Cd lines involved in Cd compartmentalization at the root level and genes expressed only in high-Cd lines acting in Cd translocation from root to shoot [40]. Two contigs annotated as aquaporins (pip1-2-like and aquaporin 1-5) were found to be expressed only in high-Cd lines and not in low-Cd lines, facilitating water transport, and hence Cd translocation. On the other hand, comparative analyses showed that the genes annotated as *NAS*, nicotianamine amine transferase (*NAAT*) and deoxy-mugineic acid synthase (*DMA*), involved in the biosynthesis and regulation of the phytosiderophores mugineic acid and NA were co-expressed and highly induced by Cd stress only in the low-Cd lines. Moreover, the contigs corresponding to the *bHLH29* and *bHLH38* transcription factors were co-expressed with *NAS*, *NAAT* and *DMA* putative genes and the Cd transporter *IREG2* [40].

Wheat reacts to cold stress by expressing various kind of adaptive responses due to the intrinsic high variability of the cold tolerance trait, shown differently by winter and spring varieties [64]. Transcriptome studies on flag leaves of the CBW0101 durum spring variety under cold stress, previously shown to have a moderate tolerance to low temperatures, were recently performed [41]. These analyses highlighted the reprogramming of gene expression patterns of CBW0101 spring variety, resembling the expression of some cold response mechanisms observed in winter wheats, with DEGs mainly involved in photosynthetic activity, lipid and carbohydrate synthesis and accumulation of amino acids and seed proteins [41]. Moreover, this transcriptomic investigation underlined the participation of long non-coding RNA in response to low temperature, unraveling the complexity of cold tolerance mechanisms in durum wheat.

The rise of atmospheric carbon dioxide (CO_2_) is part of the global climate change threat, combined with the increase in mean surface temperature and consequent water stress. Wheat responses to elevated CO_2_ and moderate high temperatures and to the interaction of both factors were recently studied [32]. The study reported that elevated CO_2_ inhibits N assimilation, leading to a C/N imbalance in the flag leaf accompanied by an induction of secondary metabolism and a modified gene expression for hormone metabolism. Several genes participating in glycolysis, light harvesting and minor carbon metabolism were down-regulated. Moreover, the reprogramming of the mitochondrial electron transport pathway, activation of cell expansion and growth, maintenance of protein homeostasis, and ROS detoxification and protection from oxidative damage were observed in response to elevated CO_2_. High temperature was shown to repress genes for C, energy, N, lipid, secondary, and hormone metabolisms. Under the combined increases in atmospheric CO_2_ and temperature, the transcript profile resembled the high temperature profile, although elevated CO_2_ partly alleviated the down-regulation of primary and secondary metabolism genes [32].

The transcriptomic approach applied to durum wheat roots to understand the Cd accumulation and transport unveiled the complexity of the response to cadmium in this crop, which involves many molecular mechanisms and genes, encoding proteins with a variety of biological functions. This information itself contributes to explaining cadmium tolerance in durum wheat. However, integration of gene expression data with proteomics and metabolomics would be valuable in the identification of key pathways that are potentially responsible for Cd tolerance and accumulation. The transcriptional approach was useful for the development of SNP markers that would be crucial for breeders to develop salinity-tolerant durum wheat cultivars [38] and contributed to understand the different pathways involved in cold stress response in durum wheat [41]. The integration of these data with physiological and metabolomic analyses may validate and elucidate new key biological pathways involved in the response of durum wheat genotypes to salinity and cold stress.

## 5. Useful Databases for Durum Wheat Research in Transcriptomics and Functional Genomics

Genetic data analysis and interpretation for cereals, including durum wheat, used to rely on model species. *Arabidopsis thaliana* has been used for many years to study most of the plant-specific processes. Later, to better address specific questions on wheat biology, *Brachypodium distachyon* was proposed as a model species [65], until the more recent advances of high-throughput sequencing technology also allowed the generation of valuable resources for other cereal crops, producing accurate gene model annotations for crop species, such as rice, maize, and wheat. Durum wheat genetic data is now present on a wide range of databases (Table 2), mainly represented by general databases, which mostly contain sequence data for a large number of organisms (also from different kingdoms), and community-specific databases, which are those that focus on a specific set of species or plant families (e.g., cereals, legumes, etc.) or data analysis (e.g., comparative analyses, gene networks, (co-)expression, protein interactions, etc.). Among general databases, durum wheat sequence data and metadata can be retrieved from the major public sequence repositories part of the International Nucleotide Sequence Database Collaboration (INSDC) including the National Center for Biotechnology Information (NCBI), the European Molecular Biology Laboratory-European Bioinformatics Institute (EMBL-EBI), and the DNA Data Bank of Japan (DDBJ). Raw sequencing data are available in GenBank, SRA and Gene Expression Omnibus (GEO) for NCBI, Arrayexpress and European nucleotide archive (ENA) for EBI-EMBL, and DDBJ Sequence Read Archive (DRA) for DDBJ [66,67,68,69,70]. Major DNA sequence databases, either general or community-specific, normally host a collection of analytical, visualization, and query tools, such as BLAST for discovering sequence similarity in big datasets, and genome browsers to interactively visualize genomic data annotations. Other public major databases supplying durum wheat genetic data are EnsemblPlants [71] which, among their numerous features store variant loci, gene models and functional annotation, and PLAZA [72], a platform for comparative, evolutionary, and functional genomics providing data resources at the DNA and protein level and several analysis tools. Among its set of tools, PLAZA provides the Integrative Orthology Viewer, which integrates several data types and methods for robust orthology computation, a valuable help in transferring gene function information across species, in particular when dealing with complex polyploid species such as durum wheat. Triticeae-Gene Tribe [73] also helps with orthology inference across complex genomes, being centered on Triticeae species, exploiting collinearity to address complex evolutionary questions within the Triticeae tribe. PLAZA and Triticeae-Gene Tribe allow functional enrichment analysis, which is a necessary step in transcriptomic data analysis. Another relevant database containing durum wheat information is Gramene [74] which focuses on genomics, comparative analyses, and variation, also providing features such as (differential) gene expression data for durum wheat close relatives (e.g., *T. aestivum* and *Hordeum vulgare*). Moreover, Gramene includes the Plant Reactome database, which stores information on plant pathways (e.g., metabolic, transport, developmental) that are projected from the curated data from rice, through orthology on more than one hundred species including durum wheat. Another important database is GrainGenes [75], which has been used for a long time to compare genetic maps (e.g., identify syntenic blocks, improve marker density around a gene of interest), and stores data on several durum wheat panels, including passport, population structure, and variant data. The need to efficiently integrate different types of omics for discovery purposes recently led to the development of Triti-Map [76], a pipeline aiming at leveraging omics data (e.g., including genomes, transcriptomes, genetic variation, and epigenomes) for gene mapping in Triticeae species. 

Although omics resources are steadily increasing for durum wheat, a larger amount of information, resources, and databases has been released and is available on bread wheat, due to its greater economic value. This is exemplified by expression browsers, which aim at making transcriptome sequencing data more accessible and usable by the scientific community. Expression browsers leverage the rising number of transcriptomic data produced and allow gene expression profile exploration in several growth stages, organs, and/or tissues of interest. Currently, the browsers incorporating wheat expression data are WheatExp, expVIP, and Wheat eFP [79,80,81], although almost all transcriptome information they contain comes from hexaploid wheat. Other developed resourceful platforms for bread wheat leveraged omics data are for advanced applications such as functional, co-expression, and knowledge network analyses [82,83,84]. One notable example is KnetMiner [84], which combines multiple types of data into knowledge networks efficiently summarizing heterogeneous but interconnected datasets. With this strategy the complexity of exploratory analyses is vastly reduced and gene function prediction and gene prioritization are made easier. Databases will continue to play an essential role for storing, organizing, accessing, and analyzing increasing amounts of biological data. The databases here described represent fundamental resources for the scientific community working on basic and applied wheat biology.

## 6. Comparative and Integrative Approaches for Data Exploitation

**Figure 2:**
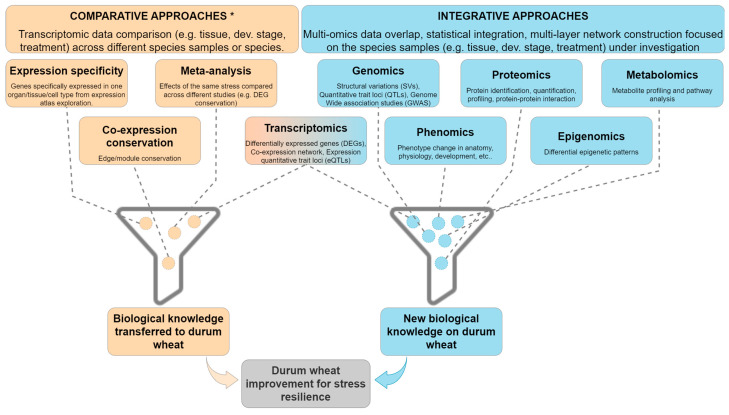
Comparative and integrative approaches for durum wheat transcriptomic data integration and exploitation. *: For these approaches orthology information is needed. Dev: developmental.

Gene expression data can be used to deepen knowledge of gene functions, biological pathways, and evolution. Advanced approaches that leverage transcriptomic data for functional discovery can imply (1) comparative analyses to study (and/or transfer) biological information across multiple organisms or (2) integrative analyses that use multiple layers of biological information (multi-omics) (Figure 2). The former approaches rely on comparing expression profiles, not only within a single organism or species [87], but also across species. For cross-species comparison of transcriptomic data, a necessary requirement is the orthology information. For this task, retrieving high quality orthologs from databases integrating several types of orthology detection methods is a valid strategy to deal with complex one-to-many and many-to-many orthologous relationships. For example, among the databases cited in the previous section, both PLAZA and Triticeae-GeneTribe store genome, gene annotation, and orthology information for the previously mentioned model species *A. thaliana* and *B. distachyon*, and for major cereal species, such as bread wheat, barley, rice, and maize, for which abundant transcriptomic resources (and other omics datasets) are available.

Among the comparative approaches, one method consists in analyzing gene expression profiles in transcriptomic datasets across specific tissues, organs, treatments, or developmental stages, in order to investigate expression conservation or divergence (expression specificity). For example, a comparative study on Poaceae species [88] highlighted that a large fraction of the orthologs with conserved expression across *Brachypodium*, sorghum, and rice were involved in physiologically similar reproductive tissues, while orthologs with divergent expression were found in flowers and seeds. Lineage-specific expression divergence (LED) applied to *Brachypodium*, rice, and sorghum found LED to be associated with processes displaying higher evolutionary rates such as domestication, male reproduction, and host-pathogen defense [89].

Gene expression profiles can also be analyzed across organisms through transcriptomic meta-analyses [90,91]. This approach, usually applied to explore conserved sets of DEGs, integrates results from independent but related studies in order to increase statistical support and generalize results. This brings into discovery sets of co-regulated genes, strengthening conclusions on pathways and processes involved. Comparative analyses of transcriptomes across wheat species or durum wheat cultivars with differences in stress resilience is a common strategy for discovering genes involved in the stress response. For instance, a different transcriptional responsive strategy for drought and heat stress was found by comparing transcriptomes of two durum wheat cultivars with contrasting water use efficiency [30,31]. In a recent study, DEGs in response to N treatments and conserved within and across species were used as a dimension reduction approach for machine learning [92]. The authors showed that using these evolutionary conserved N-responsive genes brings an improved prediction of NUE traits from transcriptomic data. By using this approach, transferable to other species and traits, the authors validated candidate transcription factors for NUE in Arabidopsis and in maize.

Another approach is to gain robust information from expression data leverages network biology and requires co-expression analyses aiming at grouping genes by their expression profiles (co-expressed genes) forming gene modules. Genes grouped in modules tend to be co-regulated or functionally related and can be compared to evaluate potential functional conservation or divergence across species [92,93], even if distantly related [94,95]. Co-expression network comparison represents an opportunity to transfer information to the durum wheat crop from historically better studied species such as rice, barley, and bread wheat, for which more (and not only transcriptomic) resources and studies exist due to their greater economic importance. In this scenario, durum wheat network modules could be improved by networks of better studied cereal species. Gene interaction information, in fact, has been used to enrich knowledge on crop species networks starting from model species [96,97]. For example, this approach led to the identification of N-regulated transcription factors targeting the same genes and network modules in Arabidopsis and rice [97]. Moreover, it was also demonstrated that using conserved gene modules, implying potential functional conservation of the orthologous gene part of these modules [98,99], leads to the transfer of more biological information across species [100]. Principles relying on co-expression such as guilt-by-association can be applied to identify new gene functions for unknown genes by analyzing the functions of the genes co-expressed with them [101,102]. This can lead to the identification of new candidate genes for a specific trait, which will be often necessary to prioritize through the gene prioritization phase by ranking the newly identified candidates after further annotation and integration of prior knowledge from diverse data sources [103]. On the other hand, integrative approaches involve the use of data from multiple levels, so called “multi-omics”, combining individual omics information, in a sequential or simultaneous manner, to understand the interplay of molecules. They help in assessing the flow of information from one omic level to the other, thus bridging the gap from genotype to phenotype [104]. Among the studies performed in durum wheat, some carried out an integration of the transcriptomic data studying variations at additional levels such as genome, epigenome, proteome, and metabolome, obtaining results with higher precision, better accuracy, and greater statistical power. Combination of genomics and transcriptomics has been used to better understand the mechanisms underlying quantitative traits. QTL, especially if stable across environments and populations, can be integrated with transcriptomic datasets to narrow down lists of candidate genes located in genomic regions associated to specific traits of interest [10]. A multi-omics approach, including genomics, revealed the control of the stem solidness by the copy number variation of *TdDof* gene in durum and bread wheat [105]. Transcriptomics is frequently applied in durum wheat genomic studies providing great potential for future innovation for the wheat scientific community and the breeding sector [5]. Moreover, data integration of both genomics and transcriptomics allowed the identification and characterization of durum wheat fungal disease resistance genes and QTL [8,11,12]. A specific type of QTL, the expression QTL (eQTL), that represents the genomic loci controlling gene-expression differences, involves the use of genomic variation and expression data, and has been used in bread wheat to identify cis- and trans-acting variants, explaining differential expression across homoeologous genes [106]. In another study, the addition of transcriptomic and genomic data with image-based phenotyping suggested that introducing wild alleles into elite durum wheat can facilitate greater phenotypic plasticity and the potential to enhance the resistance to environmental stresses [22]. Epigenomic data integration can elucidate gene expression modulation or contribute to predict robust transcription factor-target interactions due to information on methylation status and chromatin accessibility. Integration of transcriptomics and ChIP-seq data has been used to study the expression of transposable elements in response to epigenetic modifications of histones H3K27me2 and H3K27me3 [107]. A combination of transcriptomic and metabolomic approach showed recently a broader and stronger response to N starvation of durum wheat in comparison to the domesticated emmer wheat. The correlation-based networks applied, considering differential expressed genes and metabolites, displayed tighter regulation of metabolism in durum wheat than in emmer [34]. Transcriptomics and metabolomics were essential in the characterization of *T. turgidum* mutants and transgenic plants aiming at discovering the crucial role of *KAT-2B* gene in grain quality, as well as the role of jasmonic acid in grain weight determination [108]. Furthermore, metabolomics also aggregated more information to transcriptomic data allowing the finding of an association between the inhibition of leaf nitrate assimilation and the photosynthetic acclimation in durum wheat plants growing with low nitrate supply [109]. On the other hand, transcriptomic data together with proteomic analyses provided insights on the effect of pigment content in the molecular response of durum wheat to drought [17]. A large-scale multi-omics study that analyzed the transcriptome, proteome and metabolome of wheat plants infested by the wheat stem sawfly revealed the involvement of the phenylpropanoid and pentose phosphate pathways in plant defense against the insect infestation [110]. The era of large scale biological datasets entails a vast number of possibilities still unexplored in durum wheat, which will expand the opportunities both for comparative and integrative approaches. Transcriptomics can be combined also with proteomics and phospho-proteomics to better understand the role of regulators and for clarification and identification of phosphorylation sites and signaling pathways [111]. Protein phosphorylation is in fact the most frequent post-translational modification in plants in response to pathogens and was used for example in bread wheat to study interactions with *Septoria tritici* [112]. Moreover, recently, transcriptomics was adapted for the analysis of single cells. Single-cell RNA-Seq has deeply broadened transcriptomic research opportunities and goals, allowing cell-type specific exploration of gene expression profiles and pathways. For example, a study on maize and *Setaria* demonstrated that the expression of maize SHORT-ROOT (*SHR*) orthologs is specific to endodermis cell clusters, differently to what is expected from Arabidopsis, where it is expressed in the stele [113]. This allowed, along with other evidence from *shr* mutant and SHR protein cell-to-cell movement, new hypotheses on the function of SHR gene in monocots [113].

Transcription retains a leading role for the understanding of gene functions and the discovery of biological pathways involved in plant evolution and crop improvement [114]. Transcriptomic technologies are growing rapidly and their progress in terms of sensitivity and cost-effectiveness has resulted in a great expansion of gene expression publicly available data [2]. This scientific panorama now presents new perspectives on both basic and applied plant biology. In this context, databases storing and classifying huge amounts of biological information play an unprecedented and crucial role for the scientific community and will need exceptional maintenance and curation to ensure optimal performances and data completeness and consistency. The transcriptomic resources, here collected and summarized for durum wheat, could be exploited for large-scale data analyses with the exciting potential of generating new insights into gene functions and pathways involved in stress resistance. Comparative transcriptomics approaches will be crucial in transferring such biological information, in terms of conserved gene functions and unraveling functional ortholog discovery from better studied species (e.g., bread wheat and other cereals with greater economic importance). This will accelerate molecular breeding strategies aimed at improving durum wheat resilience to climate change. On the other hand, transcriptomic research and application of multi-omics data integration performed so far in durum wheat has allowed and will permit the identification of many promising gene candidates playing important roles in the context of biotic and abiotic stress responses. Regardless of the path that will lead to new knowledge in durum wheat, it will be essential to functionally validate new candidate genes using reverse genetics approaches, such as the gene knockdown or overexpression, as performed, for example, for a durum gene involved in salt and drought tolerance [115], or using genome editing technologies, employed successfully in durum wheat with high transformation efficiency [116]. The development of SNP markers for durum wheat cultivars was often a goal of RNA-seq approaches in the studies here analyzed. These markers could be used in molecular breeding programs such as Marker-Assisted Selection (MAS), which would allow durum wheat breeding, avoiding time-consuming and costly conventional breeding, for improving biotic and abiotic stress tolerance in elite durum wheat cultivars and to enrich them with novel tolerant genes.

## Figures and Tables

**Table 1:** A summary of transcriptomic studies in durum wheat plants under biotic and abiotic stresses.

Stress Type	Tissue	Developmental Stage	Platform	Ref.
**Biotic stress**				
*Fusarium graminearum*	S	50% anthesis	RNA-seq	[8]
	S	Mid-anthesis	RNA-seq	[9]
	S	Early anthesis	RNA-seq	[10]
*Blumeria graminis*	L	2-leaf seedlings	BSR-seq	[11]
*Claviceps purpurea*	O	Nearly anthesis	RNA-seq	[12]
*Rhopalosiphum padi*	L	11-day-old plants	RNA-seq	[13]
* R. padi* and *Spodoptera littoralis*	L	11-day-old plants	RNA-seq	[14]
*R. maidis*	L	2-leaf seedlings	RNA-seq	[15]
**Abiotic stress**				
Drought/water stress	FL	Zadoks 70	Affymetrix GeneChip WGA	[16]
	FL	Anthesis	Affymetrix GeneChip WGA	[17]
	FL/G	5 DPA	RNA-seq, sRNA-seq, degradome-seq	[18]
	R/L	Zadoks 22–24	sRNA-seq	[19]
	R	7-week-old plants	RNA-seq	[20]
	R	7-week-old plants	RNA-seq	[21]
	R	Zadoks 11	RNA-seq	[22]
	GL	7 DPA	Affymetrix GeneChip WGA	[23]
Heat	SD	Zadoks 21-22	RNA-seq	[24]
	G	Early grain filling	RNA-seq	[25]
	FL/G	Reproductive stage, 5 DPA	RNA-seq, sRNA-seq, degradome-seq	[26]
Drought and heat	SH	Young seedlings	sRNA seq	[27]
	G	5, 15, 25, 35, and 45 DPA	RNA-seq, sRNA-seq, degradome-seq	[28]
	FL	5, 15, 25, 35, and 45 DPA	RNA-seq, sRNA-seq, degradome-seq	[29]
	FL	Booting and tillering	Affymetrix GeneChip WGA	[30]
	L	Fully expanded mature third leaf	sRNA-seq	[31]
Heat and elevated CO_2_	FL	Zadoks 59	RNA-seq	[32]
Nitrogen starvation	R/L/FL/S	Zadoks 77	RNA-seq	[33]
	L	Zadoks 12–14	RNA-seq	[34]
	R/L/FL/S	Zadoks 77	sRNA-seq	[35]
	R/L	Zadoks 14	sRNA-seq	[36]
	SD	3-week-old seedlings	sRNA-seq	[37]
Salinity	L	Zadoks 13	RNA-seq	[38]
Cadmium	R/SH	Tillering	RNA-seq	[39]
	R	Tillering	RNA-Seq	[40]
Cold	FL	Zadoks 55	RNA-seq	[41]
**Pool of stresses**	Pools	Pools	RNA-seq, sRNA-seq	[5]

Abbreviations: DPA, days post-anthesis; HPI, hours post-inoculation; sRNA, small RNA; WGA, Wheat genome array; R, root; L, leaf; FL, flag leaf; S, spike; G, grain; O, ovary, SH, shoot; SD, seedling; GL, glume.

**Table 2:** Relevant databases for durum wheat research in transcriptomics and functional genomics. URL access date for all databases was 20 February 2022.

Database	Brief Description	URL	Ref.
SRA ENA DRA	Sequence read archives (shared) across NCBI (for SRA), EMBL-EBI (for ENA) and DDBJ (for DRA)	https://www.ncbi.nlm.nih.gov/sra https://www.ebi.ac.uk/ena/browser/ https://www.ddbj.nig.ac.jp/dra/index-e.html	[66] [67] [68]
GEO ArrayExpress	Gene expression and functional genomics data in NCBI (GEO) and EMBL-EBI (ArrayExpress)	https://www.ncbi.nlm.nih.gov/geo/ https://www.ebi.ac.uk/biostudies/arrayexpress	[69] [70]
EnsemblPlants	Integrative resource for genome-scale information and comparative genomics	https://plants.ensembl.org/index.html	[71]
PLAZA	Platform for comparative, evolutionary, and functional plant genomics	https://bioinformatics.psb.ugent.be/plaza/	[72]
Triticeae-GeneTribe	Homology inference, micro- and macro-collinearity, gene enrichment analyses	http://wheat.cau.edu.cn/TGT/	[73]
Gramene	Resource for plant comparative genomics and pathway analysis	https://www.gramene.org/	[74]
GrainGenes	Repository for a broad range of data such as genomes, variation, traits, and genetic maps	https://wheat.pw.usda.gov/	[75]
Triti-Map	Multi-omics data integration for locating causal variants and candidate genes.	http://bioinfo.cemps.ac.cn/tritimap/	[76]
WheatOmics	Multiple omics data and tools for functional genomics studies in wheat	http://wheatomics.sdau.edu.cn/	[77]
Wheat-SnpHub	Large-scale wheat genomic variation data retrieval, analysis, and visualization	http://wheat.cau.edu.cn/Wheat_SnpHub_Portal/	[78]
WheatExp expVIP * Wheat eFP *	Expression browsers incorporating RNA-seq data on different stages, tissues/organs, and treatments	https://wheat.pw.usda.gov/WheatExp/ http://www.wheat-expression.com/ https://bar.utoronto.ca/efp_wheat/cgi-bin/efpWeb.cgi	[79] [80] [81]
WheatNet *	Genome-scale functional gene network and gene prioritization analyses for bread wheat	https://www.inetbio.org/wheatnet/	[82]
WheatCENet *	Comparative co-expression network analysis for *T. aestivum*, *T. dicoccoides*, *T. urartu* and *Ae. tauschii*	http://bioinformatics.cau.edu.cn/WheatCENet/	[83]
KnetMiner *	Integrated and interactive gene and gene network discovery and prioritization	https://knetminer.com/	[84]
T3/Wheat *	Database schema to combine, visualize, and interrogate phenotype and genotype data	https://wheat.triticeaetoolbox.org/	[85]
CerealsDB *	Information on SNPs and traits in the genomes of bread wheat and its relatives for breeding purposes	https://www.cerealsdb.uk.net/cerealgenomics/CerealsDB/indexNEW.php	[86]

Abbreviations: NCBI, National Center for Biotechnology Information; DDBJ, DNA Data Bank of Japan; EMBL-EBI, European Molecular Biology Laboratory-European Bioinformatics Institute; SRA, Sequence Read Archive; ENA, European Nucleotide Archive; DRA, DDBJ Sequence Read Archive; GEO, Gene Expression Omnibus. *: No data on durum wheat are incorporated.

## Data Availability

Not applicable.

## Supplementary Materials

The following supporting information can be downloaded at: https://www.mdpi.com/article/10.3390/plants12061267/s1, Table S1. List of transcriptomic studies with publicly available data (i.e., deposited in SRA or ArrayExpress) on durum wheat. For studies including multiple species/subspecies, only durum wheat genotypes are presented. NA: Not applicable or not available data; DPA: days post-anthesis; HPI: hours post-inoculation; var.: variety; cv(s): cultivar(s); N: Nitrogen; DS: Drought stress; HS: Heat stress; SWC: Soil Water Content; WL: Water-Limited; FC: Field Capacity
